# The Prevalence of Anxiety and Depression in Chinese Asthma Patients

**DOI:** 10.1371/journal.pone.0103014

**Published:** 2014-07-23

**Authors:** Shigang Liu, Ruohan Wu, Lei Li, Li Liu, Guoqin Li, Xia Zhang, Yuyan Guo, Yinghui Wang, Hong Zhang, Guangxi Li, Hui Li

**Affiliations:** 1 Guang'anmen Hospital, China Academy of Chinese Medical Sciences, Beijing, China; 2 Beijing University of Chinese Medicine, Beijing, China; 3 Pulmonary and Critical Care Medicine, Mayo Clinic, Rochester, Minnesota, United States of America; University of Electronic Science and Technology of China, China

## Abstract

It is well documented that the psychiatric disorders are common in Asthma patients in China while the studies on the relationship between asthma control and psychological disorder are comparatively rare. We therefore performed a cross-sectional study on asthmatic outpatients in one Chinese tertiary center to investigate the correlation among asthma, anxiety and depression. Demographic data, anxiety and depression scores, the level of asthma control were collected in 261 patients. All patients were evaluated with Asthma Control Test (ACT), Self-Rating Anxiety Scale (SAS) and Self-rating depression scale (SDS) questionnaires. A multivariate analysis was performed to investigate the relationship between asthma control and psychological disorder. The result showed that 31 asthma patients had anxiety symptom while thirty-five asthma patients had depression. One hundred and ninety-two asthma patients were well controlled and 69 patients were not. The study found a negative correlation between ACT and SAS(r = −0.231, p<0.001) as well as ACT and SDS(r = −0.23, p<0.001) and depression (OR: 12.295, 95%CI: [5.374–28.128], p<0.001) were both independently associated with poor asthma control. We concluded that Asthma control is greatly affected by psychological disorder in Chinese patients.

## Introduction

According to WHO estimates, 235 million people suffered from asthma worldwide and 20 million in China. Asthma is not just a public health problem for high income countries but occurs in all countries regardless of the level of development. Over 80% asthma death occurs in low and lower-middle income countries [1]. Asthma is not just a local airway inflammation but also highly affected by cholinergic nerve system [2]. Some researchers already observed that asthmatic subjects had hyper-reactive *α*-adrenergic responsiveness and hypo-reactive *β*-adrenergic responsiveness [3], [4]. Several epidemiology studies also consistently documented that anxiety and depression were prevalent in patients with asthma, and associated with more exacerbations and increased health care utilization [5], [6] in many countries. Several studies also showed that placebo effect can be clinically meaningful to asthma patients which indicated that asthma attack might have certain correlation with emotional [7], [8].

There are about 30 million asthma patients in China. The asthma control rate improved dramatically after the implementation of GINA (Global Initiative for Asthma) all over the country. Nowadays, much less patients were admitted to the emergency room or hospital ward for asthma attack. However, there are still quite a large proportion of asthma patients who only remained partial control. Some Chinese researchers had proposed that emotion fluctuation might be one of the most important factors contributing to the poor asthma control [9], [10]. They also showed that the existence of psychiatric comorbidities could predict the future risk of asthma exacerbation [11]. One study from China already documented that anxiety and depression were more common in asthmatic patients when compared to healthy controls [12]. While the report on the relationship among anxiety, depression and asthma control are still comparatively rare in China. Our study aims to explore the relationship between asthma control and psychological disorder in Chinese population by measuring the Self-Rating Anxiety Scale (SAS) [13], Self-rating depression scale (SDS) [14] and asthma control test (ACT) [15]. We hypothesized that the depression and anxiety are two important independent risk factors for uncontrolled asthma.

## Materials and Method

### Study subjects

Male and female patients aged from 18 to 79 with asthma as diagnosed and managed according to GINA 2012 [16] in Guang'anmen Hospital from June to September 2012. Exclusion criteria included as following: patients without the standard care according to GINA, patients with asthma attack; alcohol or drug abuse; women who were pregnant or breastfeeding; patients with family history of mental illness; patients with severe chronic co-morbidities such as cardiovascular, liver, kidney, nerve, blood, or severe tumor. All patients provided written informed consent prior to participating in the study. The study was approved by Guang'anmen Ethic Institutional Review Board. No minors/children were enrolled in this study. Only patients who provided written informed consent prior to participating in the study were enrolled. The approval number is 2014EC001-01.

### Measurement of asthma control, anxiety and depression

All patients were required to finish all the ACT, SAS and SDS questionnaires at the beginning. ACT questionnaire includes five questions to examine the asthma severity. There are 5 questions in ACT questionnaire, each question has 5 with the score ranging from 1 to 5. A total score of 25 means completely control, score between 20 to 24 is defined as well control and score less than 20 as poorly control [15].

#### Self Rating Anxiety Scale (SAS)

20 items with a four-point Likert scale. A higher score indicates more severe anxiety symptoms. Validity and reliability tested in the People's Republic of China [13].

#### Self Rating Depression Scale (SDS)

20 items with a four-point Likert scale. A higher score indicates more severe depression symptoms. Validity and reliability are also measured the People's Republic of China [14].

#### Covariates of asthma control

The following variables were examined as potential confounders of the asthma anxiety and depressive symptoms association. Sociodemographic data (age, sex, insurance type, marriage, retirement) were collected. Smoking status was classified as current, former or never smoker. Height (in cm) without shoes was measured using a vertical ruler. Weight (in kg) in light clothing was measured using a calibrated scale. Body mass index was calculated in kg/m2.

### Data collection and analysis

Continuous data were described as mean(x) and standard deviation (SD). Categorical variables were displayed as number (percentage). Continuous variables were compared using t-test and analysis of variance (ANOVA). Dichotomous variables were compared using chi-square or a Fischer exact test. A logistic regression model was used to evaluate the risk factors for poorly controlled asthma adjusted by all the covariates influencing asthma control. A multiple linear correlation regression model was used to examine the relationship between SAS, SDS and asthma control while adjusting for asthma severity, age, gender, education and other covariates, all the statistical analyses were conducted by SPSS17.0 (SPSS, Chicago, USA).

## Result

**Figure 1:**
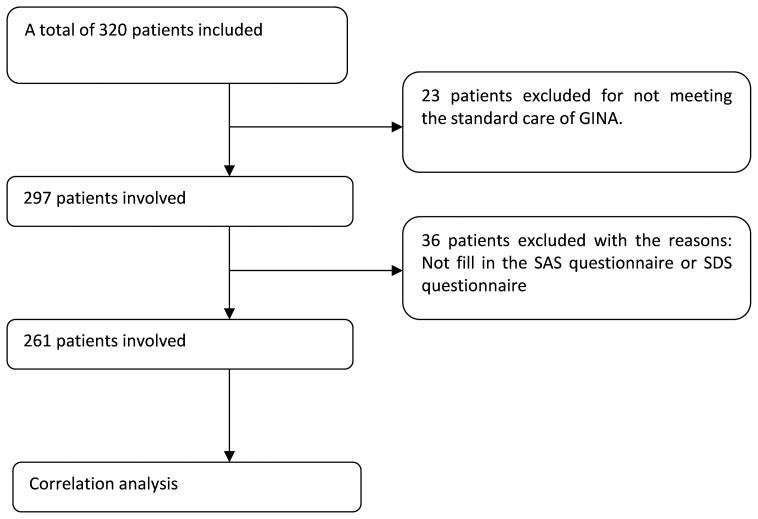
The flowchart of study enrollment.

A total of 261 patients were enrolled in the study (Figure 1). Nearly a quarter of the patients were male (24.9%). Baseline demographics and clinical characteristics were similar between well controlled asthma and poorly controlled asthma groups. One hundred and ninety-two patients enrolled in the study were considered as the well controlled asthma measured by ACT (score ≥20) (Table 1).

**Table 1:** Baseline demographics and characteristics between asthma patients with and without control.

Characteristics	Well control	Poor control	*P*
Participants, n(%)	192(73.56%)	69(26.44%)	0.003*
Age, year (m±s)	54.62±12.37	54.75±13.14	0.840
Gender, Female(n,%)	146(74.49%)	50(25.52%)	<0.05*
Marriage, n(%)	180(93.75%)	62(89.85%)	0.286
Retired, n(%)	98(51.04%)	27(39.13%)	0.090
ACT Score	22.59±1.65	16.38±2.41	<0.001*
Smoking	47(24.48%)	18(26.08%)	<0.001*
Uninsured	23(11.98%)	16(23.19)	<0.001*
Anxiety patients	12(6.25%)	19(27.54%)	<0.001*
Depression patients	9(4.69%)	26(27.68%)	<0.001*

*Significant value with p value less than 0.05.

**Figure 2:**
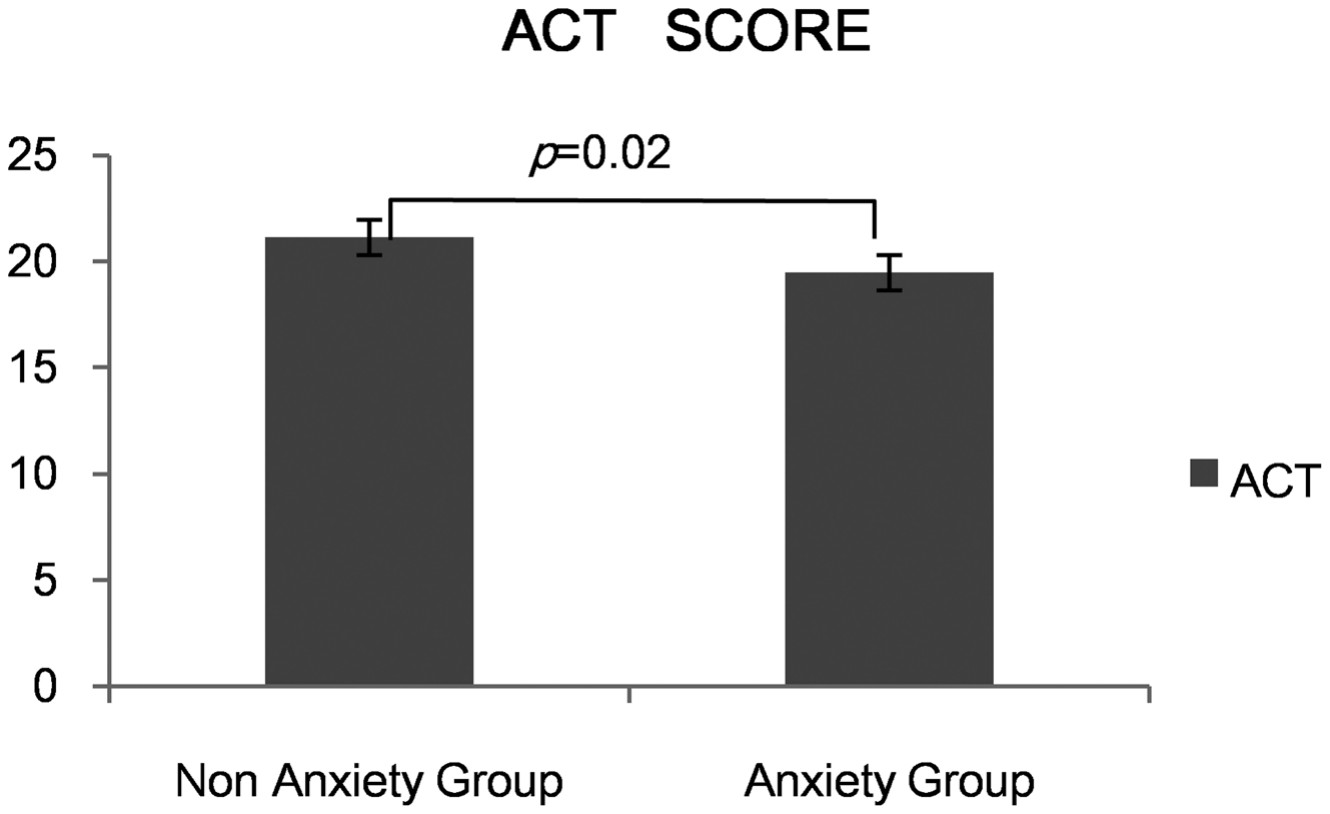
ACT score between Anxiety and Non anxiety group.

**Figure 3:**
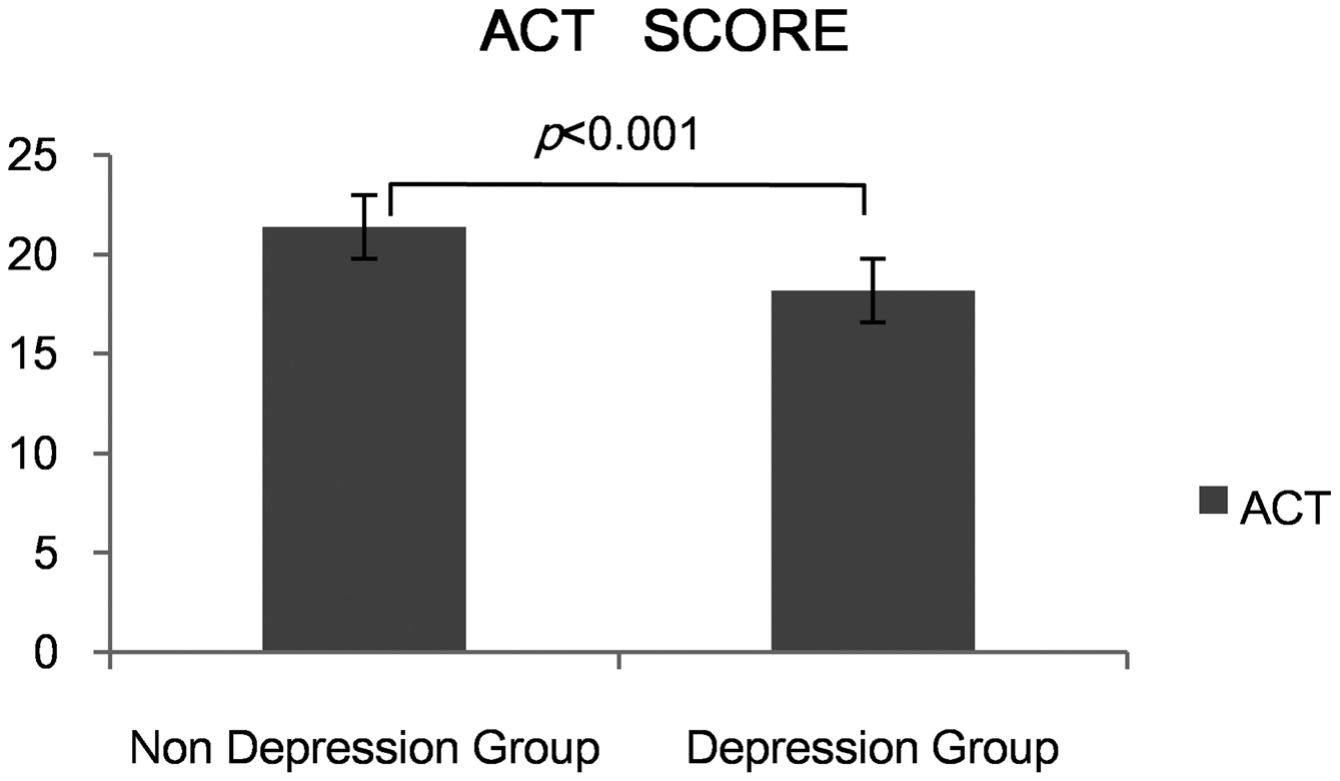
ACT score between Depression and Non depression group.

**Figure 4:**
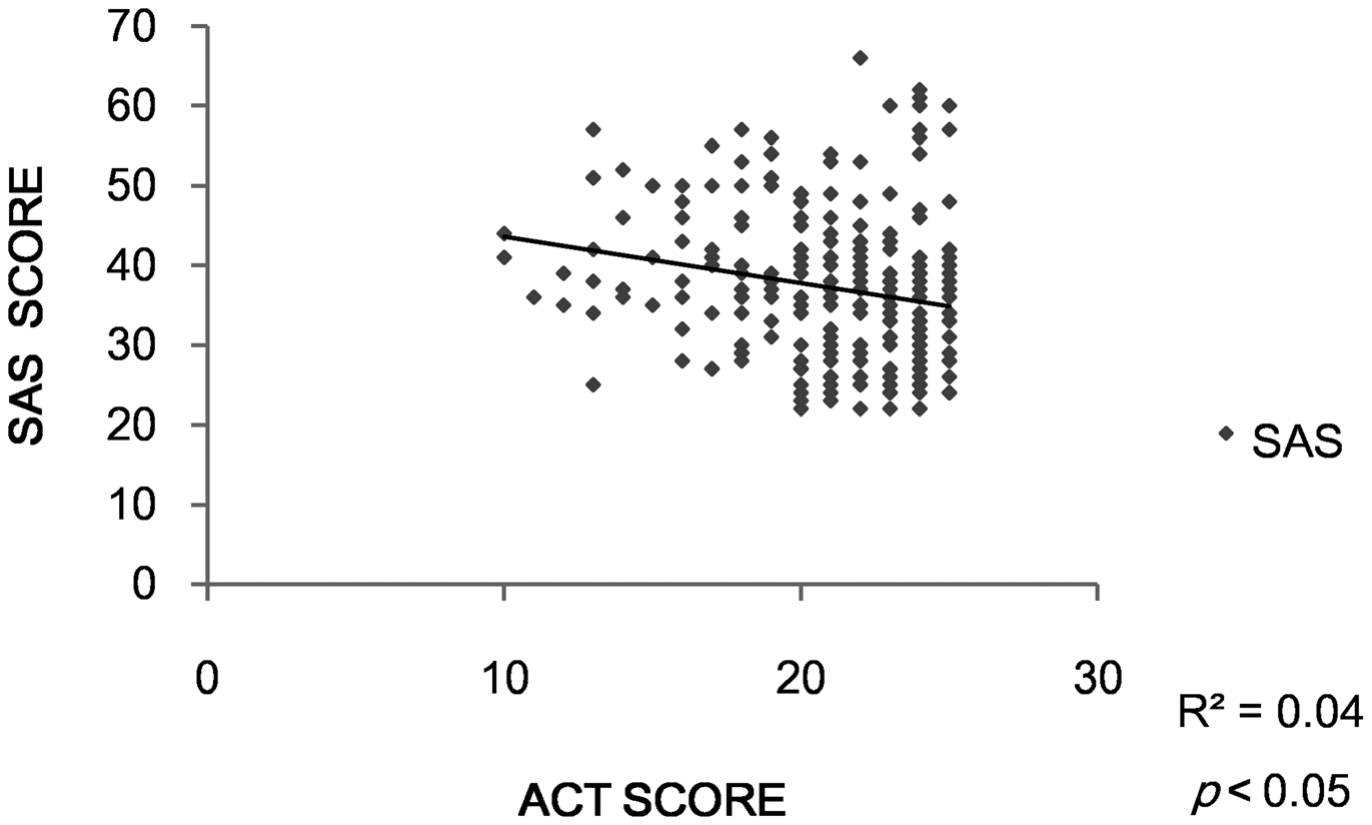
Correlation between ACT and SAS.

**Figure 5:**
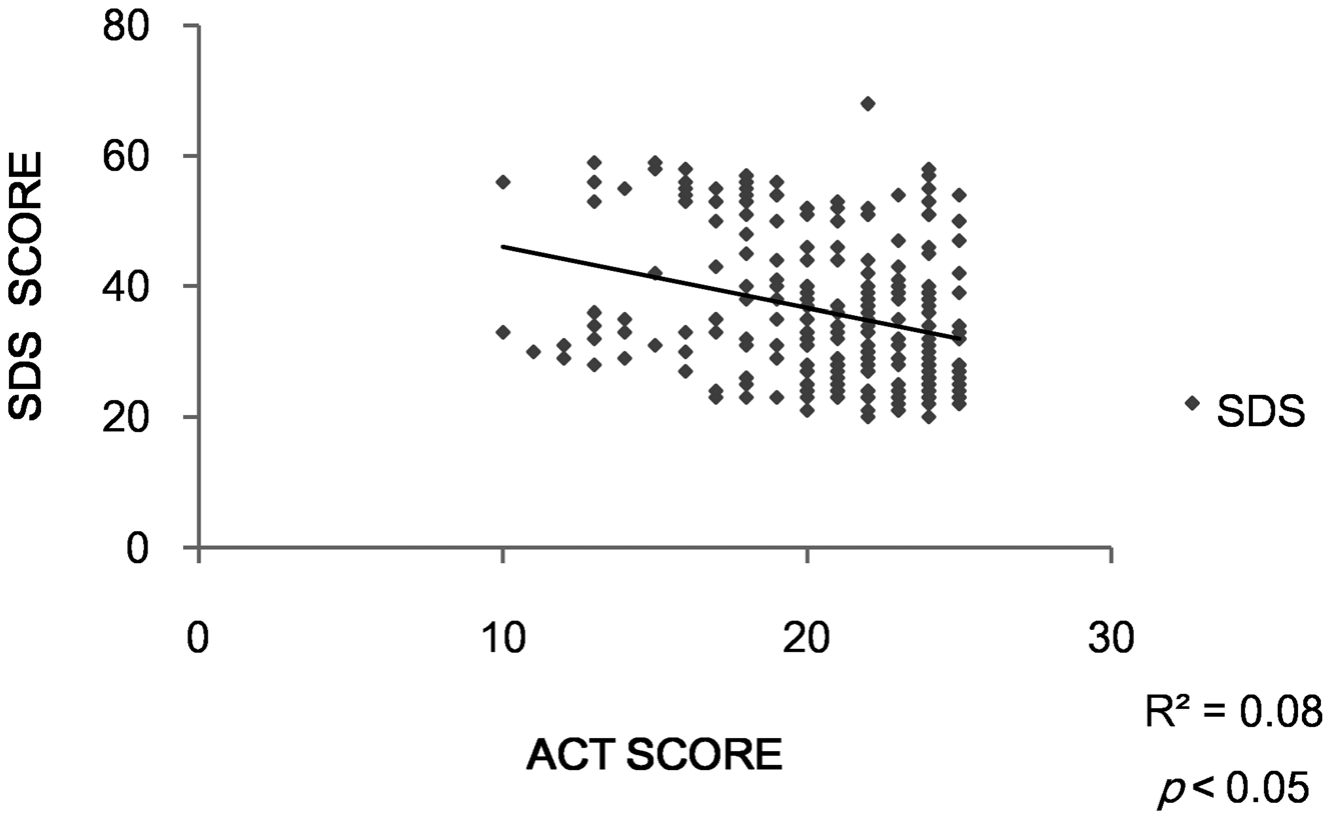
Correlation between ACT and SDS.

Thirty-one asthma patients (defined as Anxiety Group) had anxiety symptom while 35 (defined as Depression Group) presented with depression. Twenty patients had both anxiety and depression symptoms. Both the anxiety group and the depression group had lower ACT scores than the other two groups (*p*<0.05)( Figure 2, 3). We found a negative correlation between ACT and SAS (r = −0.23, *p*<0.05). The same condition happened between ACT and SDS (r = −0.28, *p*<0.05)( Figure 4,5).

**Figure 6:**
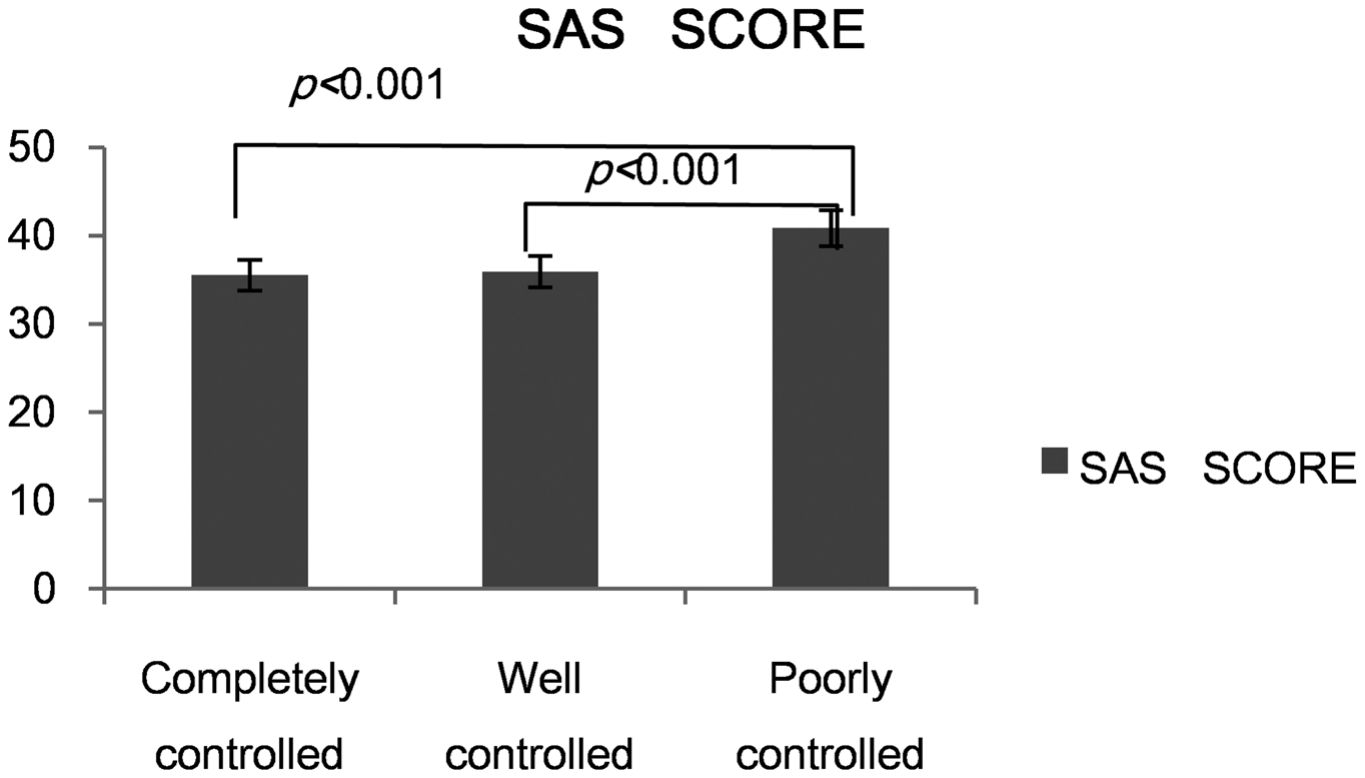
Anxiety score across different asthma groups.

**Figure 7:**
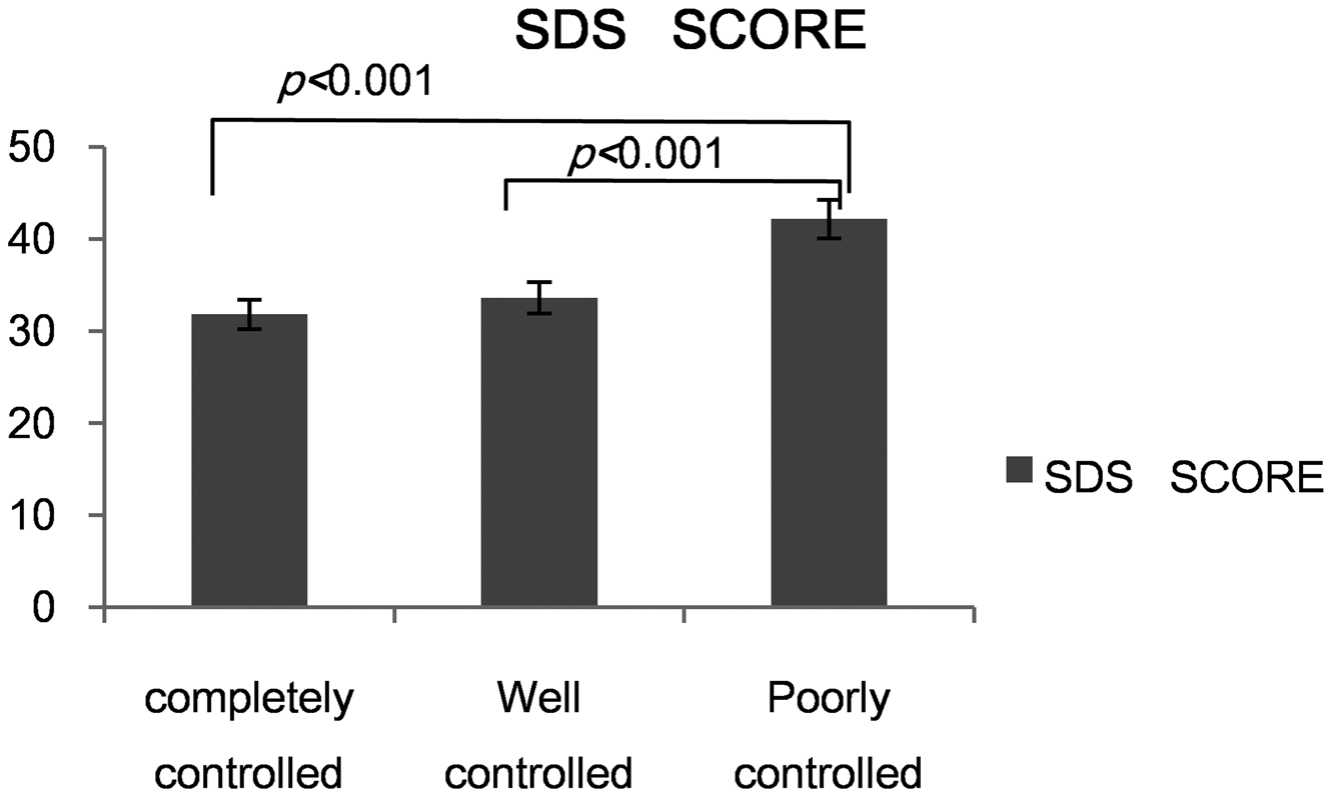
Depression score across different asthma groups.

We divided all patients into three groups according to the ACT score. The result showed that both the completely controlled group and well controlled group had a lower score than the poorly controlled group in SAS and the SDS (*p*<0.001)(Figure 6, 7).

The result of logistic regression was shown in table 2. Risk factors include female sex, age, past or smoking, uninsured, anxiety and depression. The multivariate analysis result showed that smoking, uninsured, anxiety and depression were both independent risk factors associated with poor asthma control evaluated by ACT.

**Table 2:** Multivariate analysis of poorly controlled asthma (ACT<20).

Characteristics	Odds ratio	95%CI	*P*
Female sex	0.829	0.444–1.547	0.556
Age≥60	0.763	0.422–1.377	0.369
Anxiety	4.860	2.233–10.583	<0.001*
Depression	12.295	5.374–28.128	<0.001*
Smoking	1.897	1.022–3.521	0.042*
Uninsurance	2.218	1.092–4.506	0.028*
Retired	0.617	0.352–1.080	0.091

*Significant value with p value less than 0.05.

Since there was a large range of age among participants we conducted subgroup analysis to discuss whether different age leaded to different ACT, SAS, SDS scores. The result showed no significant difference in different ages (p>0.05) (Table 3).

**Table 3:** ACT, SAS, SDS scores in different age group.

Age	ACT	SAS	SDS
≤30	21.80±2.57	35.93±6.62	34.33±7.98
31–40	21.32±3.16	38.79±10.36	38.00±11.04
41–50	20.58±3.44	38.73±9.94	39.51±12.14
51–60	21.10±3.42	37.83±9.67	35.80±11.61
61–70	30.78±3.45	34.95±7.83	32.45±9.04
≥71	20.63±3.10	37.38±10.64	35.13±10.39
X2	2.605	4.716	9.213
p	0.761	0.452	0.101

**Figure 8:**
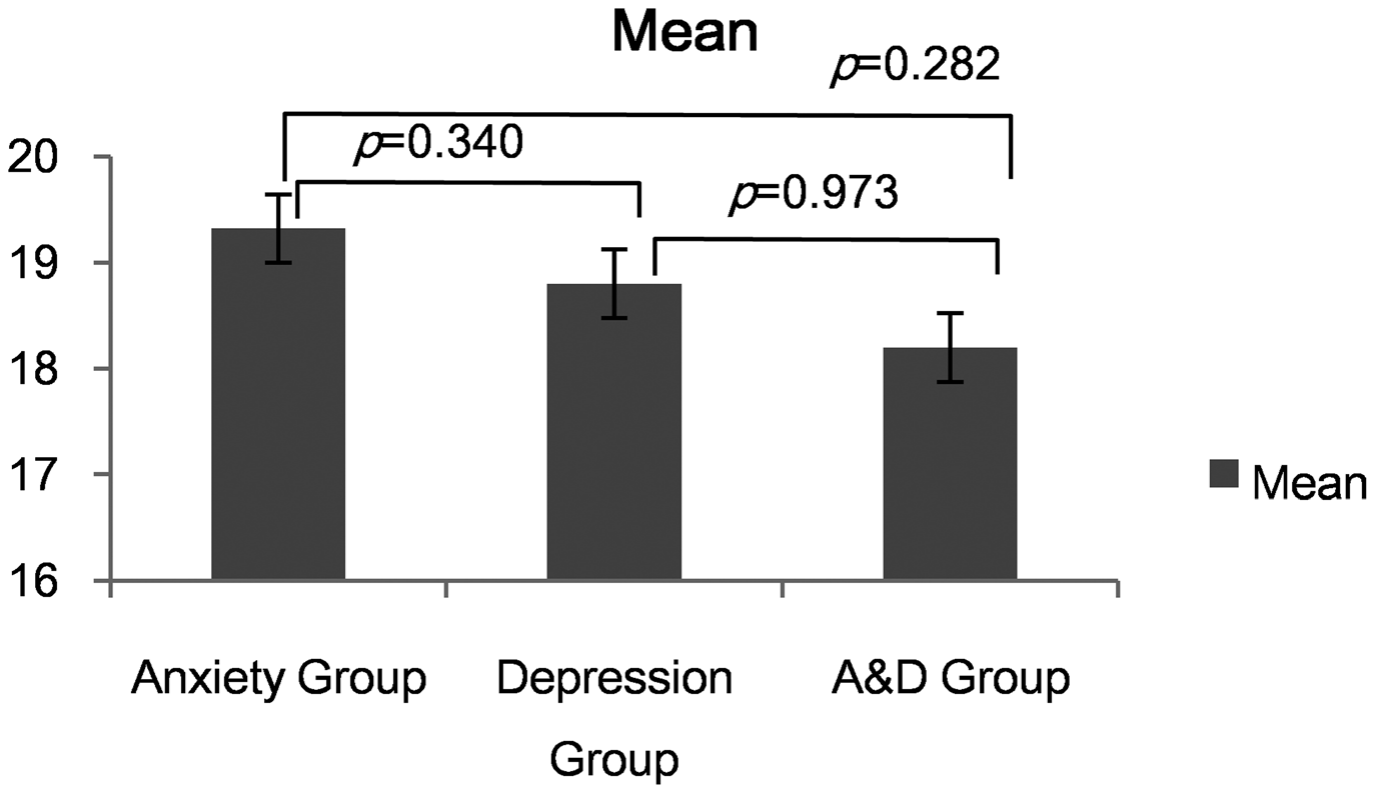
ACT Scores of Anxiety Group, Depression Group and A&D Group.

There were 31 patients had anxiety symptom, 35 patients had depression symptom while 20 patients had both symptoms. There was a trend toward worse asthma control in patients with both symptoms group although no statistical significance was reached (Table 4 and Figure 8).

**Table 4:** ACT Scores of Anxiety Group, Depression Group and A&D Group.

	Anxiety (n = 31)	Depression (n = 35)	A &D (n = 20)	Chi-Square	P
ACT Score	19.32±3.700	18.80±3.076	18.20±2.648	1.642	0.440

## Discussion

This study reported the correlation between asthma and depression in a sample of stable asthma patients from June to September in Beijing Guang'anmen Hospital. Our study showed that depression and anxiety were independently associated with asthma control. Our results were similar to the previous reports carried out in other countries [17].

Reported prevalence for depression and anxiety among patients with asthma varied in the literatures. Several studies suggest that the prevalence of psychological disorder is increasing among adults with asthma in China [18], [19]. Some researchers found higher depression prevalence [20], [21]. while others noticed more anxiety population [22], [23]. In our study, the number of patients with depression symptom was a little higher than the patients with anxiety symptom [21]. Regardless depression or anxiety, all asthma patients with psychology disorder were poorly controlled, which was in accordance with many other domestic and foreign studies [24], [25]. There were 31 anxiety patients (11.88%) and 35 depression patients (13.41%) among all 261 asthma patients lower than the studies other researchers [26] performed. It could be explained that the patients of our study were in stable stage and the asthma symptoms were not as severe as persistent stage.

Interestingly, the baseline characteristics showed that there were 6.25% well controlled asthma patients still complicated with anxiety while 4.69% had depression. To discuss the psychological effects on asthma patients some clinical researchers did researches by using Trait Coping Style Questionnaire (TCSQ), Life Event Scale (LES) and Eysenck Personality Questionnaire (EPQ). The result showed that living environment, character, personality and life style seemed to trigger the exacerbation of asthma [27], [28]. The potential reasons for the psychological disorders in China might come from work stress, introversion and sensitive interpersonalrelationshipin China [29].

Previous researches proved that psychological factors could lead to asthma attacks by affecting nerve, immune and internal secretion [30]. Mauser PJ showed that histamine may serve the central nervous system neurotransmitter function in reflex bronchoconstriction in guinea pigs [31], [32]. An animal experiment found that asthma rats with psychological stress had a higher level of histamine than the asthma rats without psychological stress [33], [34]. We summarized several reasons as follows for the incidence of anxiety and depression. First, lack of asthma knowledge and compliance to the therapy. During our study period we found that nearly half of the asthma patients did not take any medicine in stable phase. Second, economic burden or social pressure can cause anxiety and depression. There was about 21.74% of poorly controlled asthma patients treated their disease at their own expense which was a huge burden for them. Due to the limited health care resource, many patients need pay the medical expense out of their own pockets.

The major strength of our study was that we measured anxiety, depression scores and ACT scores at same time. We also enrolled an enough subjects into our study. We did find a significant correlation between asthma control and psychological disorder. Patients with better asthma control were less susceptible to anxiety or depression while anxiety and depression could be risk factors of asthma. The indication of our study is that we need to give patients health education and psychological counseling other than the continuous drug therapy [35], [36] to gain the perfect asthma control. Also, the patients should be positive and optimistic in facing disease challenge to achieve the best control of asthma and improve their quality of life [37].

The study had several potential limitations. First, the study was only performed in one institution and the results might not be generalizable to other places. However, the inclusion of around 300 consecutive outpatients from asthma clinic reduces the risk of patients' selection bias. Second, The SAS and SDS questionnaires both contain 20 questions and the perception of the questions might limit the reliability of the anxiety and depression measurement although both the validity and accuracy of these two instruments were tested in Chinese population [38], [39]. Finally, the nature of cross-section study limits our ability to conclude the definite causative relationship between asthma control and psychological disorder.

In summary, our study highlighted the high prevalence of anxiety and depression in the asthma patients with poorly controlled asthma reported higher rates of emotional issues. These findings confirm that the anxiety and depression are negatively associated to asthma control.
